# Supplementary material for: End Sequence Analysis Toolkit (ESAT) expands the extractable information from single-cell RNA-seq data
Source: Genome Res. 2016 Oct;26(10):1397–410. doi: 10.1101/gr.207902.116 (PMC5052061; doi:10.1101/gr.207902.116)
Supplement: Supplemental Material [file supp_gr.207902.116_Supp_Materials.tar.gz › ESAT-esat_v0.1/doc/html/classbroad_1_1core_1_1sequence_1_1_sequence_region_window.html]

ESAT: broad.core.sequence.SequenceRegionWindow Class Reference


ESAT


- Main Page
- Related Pages
- Packages
- Classes
- Files

- Class List
- Class Index
- Class Hierarchy
- Class Members

All Classes Namespaces Files Functions Variables Enumerator Pages

- broad
- core
- sequence
- SequenceRegionWindow

Public Member Functions |
Protected Member Functions |
List of all members

broad.core.sequence.SequenceRegionWindow Class Referenceabstract

|  |  |
| --- | --- |
| Public Member Functions | |
|  | SequenceRegionWindow (SequenceRegion region) |
|  | |
| abstract void | computeScore () |
|  | |
| double | getScore () |
|  | |
| SequenceRegion | getRegion () |
|  | |

|  |  |
| --- | --- |
| Protected Member Functions | |
| void | setScore (double score) |
|  | |

## Constructor & Destructor Documentation

|  |  |  |  |  |  |
| --- | --- | --- | --- | --- | --- |
| broad.core.sequence.SequenceRegionWindow.SequenceRegionWindow | ( | SequenceRegion | *region* | ) |  |

## Member Function Documentation

|  |  |  |  |  |
| --- | --- | --- | --- | --- |
| abstract void broad.core.sequence.SequenceRegionWindow.computeScore | ( |  | ) |  |

abstract

|  |  |  |  |  |
| --- | --- | --- | --- | --- |
| SequenceRegion broad.core.sequence.SequenceRegionWindow.getRegion | ( |  | ) |  |

|  |  |  |  |  |
| --- | --- | --- | --- | --- |
| double broad.core.sequence.SequenceRegionWindow.getScore | ( |  | ) |  |

|  |  |  |  |  |  |
| --- | --- | --- | --- | --- | --- |
| void broad.core.sequence.SequenceRegionWindow.setScore | ( | double | *score* | ) |  |

protected

---

The documentation for this class was generated from the following file:

- java/broad/core/sequence/SequenceRegionWindow.java


---

Generated on Fri Aug 15 2014 15:10:56 for ESAT by  

 1.8.7
